# The relationship between extracurricular sports participation and subjective well-being in junior high school students: a moderated mediation model

**DOI:** 10.3389/fpubh.2024.1456219

**Published:** 2024-08-16

**Authors:** Feifei Li, Liqiang Li, Xiaomin Du, Xiaozan Wang

**Affiliations:** ^1^School of Physical Education and Health, East China Normal University, Shanghai, China; ^2^School of Physical Education and Health, Yili Normal University, Xinjiang, China; ^3^School of Physical Education, Xizang Minzu University, Xianyang, China; ^4^Research on Physical Education for Adolescents of Shanghai Social Science Innovation Research Base, Shanghai, China

**Keywords:** subjective well-being, extracurricular sports participation, emotion regulation, physical education class participation, mechanism of action

## Abstract

**Objective:**

To investigate the direct effect of extracurricular sports participation on subjective well-being among junior high school students, and the mediating role of emotion regulation and moderating role of physical education (PE) class participation.

**Methods:**

Using data from the Program for International Student Assessment (PISA), we analyzed the extracurricular sports participation, emotion regulation, subjective well-being, and PE class participation of 113,203 junior high school students.

**Results:**

After controlling for country, gender, and health status, extracurricular sports participation significantly predicts subjective well-being. Emotion regulation mediates the relationship between extracurricular sports participation and subjective well-being. Both the direct effect of extracurricular sports participation on subjective well-being and the mediating effect of emotion regulation are moderated by PE class participation. The effect is stronger among students with high PE class participation compared to those with low participation.

**Conclusion:**

There is a moderated mediation effect between extracurricular sports participation and subjective well-being among junior high school students. Emotion regulation mediates this relationship, while PE class participation enhances the impact of emotion regulation on subjective well-being.

## 1 Introduction

Adolescence is a decisive stage in human development during which individuals can experience intense physical, psychological, emotional, and social changes (1). The Programme for International Student Assessment (PISA) is an international comparative study initiated by the Organization for Economic Cooperation and Development (OECD). The PISA 2022 assessment included a Subjective Well-Being Questionnaire (SWBQ) that collected extensive data on the subjective well-being of 15-year-old students in participating countries (2). Subjective well-being refers to an individual’s overall evaluation of their life conditions, encompassing life satisfaction, positive emotions, and negative emotions (3). It is a crucial indicator of psychological health (4). With socio-economic development, student well-being has garnered increasing attention in academic circles. Many parents now emphasize “happiness,” “kindness,” and “health” over academic achievement when asked about their expectations for their children (5). From the perspective of sociology and psychology, sports participation is an important strategy to realize children’s socialization, which is essentially a socialization process (6–8).

For middle school students, low well-being can lead to maladaptive behaviors during adolescence, trigger psychological crises, and develop into problem behaviors or antisocial behaviors (9). Studies show that physical exercise improves well-being across various indicators (10), and sports participation is considered an essential health-promoting behavior (11). However, the extent to which this positive correlation exceeds a statistical association remains controversial. Some research shows that physical activity induces positive changes in well-being (12), while other studies suggest that this relationship reflects common genetic factors influencing physical activity and well-being (13). The individual-environment interaction theory posits that behavioral problems result from the interaction of negative environmental factors and individual traits (3, 14).

Although many studies have investigated factors influencing the well-being of college and high school students, few have focused on middle school students (15). The factors influencing student well-being may differ during middle school, reflecting the transition from childhood to adolescence (16). Therefore, to elucidate the mechanisms enhancing middle school students’ subjective well-being, it is necessary to examine the mediating and moderating effects of behavioral factors (extracurricular sports participation), cognitive factors (school PE class participation), and self-regulation abilities (emotion regulation) from an integrated perspective (3). This approach aims to answer how and when extracurricular sports participation influences individual subjective well-being. Clarifying the mechanisms of extracurricular sports participation on subjective well-being is crucial for understanding its impact and has significant implications for encouraging student participation in sports and enhancing their well-being.

### 1.1 Relationship between extracurricular sports participation and subjective well-being

Subjective well-being (SWB) is a multidimensional concept that includes an individual’s happiness, overall life satisfaction, satisfaction with important life domains, and related emotional states (17, 18). Extracurricular activities are crucial for promoting well-being. Sports activities significantly impact well-being (19–21). Numerous studies report relationships between well-being and sports activities across various countries (6, 22). Research indicates that students participating in extracurricular activities experience more positive outcomes (18), including healthier self-perception, positive attitudes, and higher subjective well-being (23–25). Additionally, studies by Holder et al. (26). Suggest that active leisure (e.g., exercise or sports) is positively correlated with well-being compared to passive leisure (e.g., reading, watching movies, or using computers). Sports participation is also associated with behavioral well-being, particularly regarding positive attitudes, personality, physical and mental well-being scores, healthy lifestyles, and psychological health (27, 28).

Recent studies demonstrate that subjective well-being is related to extracurricular activity participation. For instance, certain distress and mental health indicators are associated with extracurricular activity participation (29) Positive connections with extracurricular activities contribute to students’ mental health (14, 30). Moreover, academic success and emotional well-being positively correlate with past and current extracurricular activity participation (18, 31). Furthermore, lower anxiety and depression symptoms, higher life satisfaction and optimism (18), and higher psychological health levels are associated with participation in sports and other extracurricular activities (32). Active participation in sports can reduce negative emotions like depression and anxiety and provide positive and enjoyable experiences.

Based on these findings, we hypothesize:

H1: Extracurricular sports participation positively predicts well-being.

### 1.2 The mediating role of emotion regulation

Emotion regulation refers to how individuals use strategies to manage the occurrence, experience, and expression of emotions (33). The emotion regulation theory posits (34, 35) that sports activities impact participants’ emotions in two ways: improving emotional tone immediately after training and reducing negative emotional states such as anxiety, irritability, and guilt (3, 36). Research shows that sports activities improve emotions by increasing dopamine, serotonin, and norepinephrine levels in the brain (37). High-intensity aerobic exercise is positively correlated with positive emotions (38), while moderate-intensity anaerobic exercise significantly improves emotions (39). Both aerobic and stretching/balancing exercises effectively enhance well-being (40). Physical exercise is associated with mental disorders (e.g., depression, anxiety, and stress) and psychological health (e.g., self-esteem, self-concept, self-efficacy, optimism, and well-being) (10, 41). In schools, physical activities play a crucial role in improving adolescents’ mental health and have unique advantages in promoting both physical and mental health (42, 43). School PE curriculum interventions can improve adolescents’ mental health (44). The Self-Regulatory Executive Functioning (S-REF) theory suggests that positive emotions, as a component of psychological well-being, are characterized by happiness, enjoyment, and satisfaction, reflecting overall subjective well-being (41, 45, 46). Current research on mediating variables between exercise and subjective well-being includes loneliness, self-efficacy, and motivation, with findings mostly indicating partial mediation.

Therefore, we hypothesize:

H2: Extracurricular sports participation indirectly influences individual well-being through the mediating role of emotion regulation.

### 1.3 The moderating role of PE class participation

The ultimate goal of schools is to instill self-belief in students and equip them to live happy, independent lives (47). If schools fail to achieve this, their role in society must be questioned (48), which can harm both the school and society. Schools are crucial settings for promoting adolescents’ lifelong development, mental health, and overall subjective well-being (49). However, limited attention has been given to the iterative and interactive roles of social environmental and self-system factors in predicting adolescents’ subjective well-being, especially in the school context. The self-system model of motivational development categorizes students’ participation in school activities into four parts: context, self, action, and outcome (50). Context represents environmental aspects (e.g., school) that create positive conditions and support; self, or self-system, includes psychological needs for competence, autonomy, and relatedness; action refers to students’ participation in educational processes; and outcome includes the cumulative results of context and self-system influences on participation (49). This model suggests that self-system variables mediate the relationship between context and participation-related actions (51, 52). Therefore, school PE education provides opportunities for students to participate in sports, motivating them to engage in extracurricular activities. Some scholars believe subjective well-being is similar to the emotional dimension of participation, sharing common characteristics (49, 53). Additionally, personal factors like self-cognition influence the relationship between contextual factors and adolescents’ subjective well-being (49, 54, 55). Academic learning and social interaction are key activities in adolescents’ school life. Self-cognition in these areas significantly impacts psychological health indicators (e.g., subjective well-being and emotion regulation). According to the self-system model, extracurricular sports and PE class participation reflect the perceived satisfaction of needs for competence and relatedness. Therefore, the principles of the self-system model can also be applied to subjective well-being (49).

Based on this, we hypothesize:

H3: The number of days students participate in PE classes per week moderates the relationship between extracurricular sports participation and well-being, and the mediating effect of emotion regulation.

**Figure 1:**
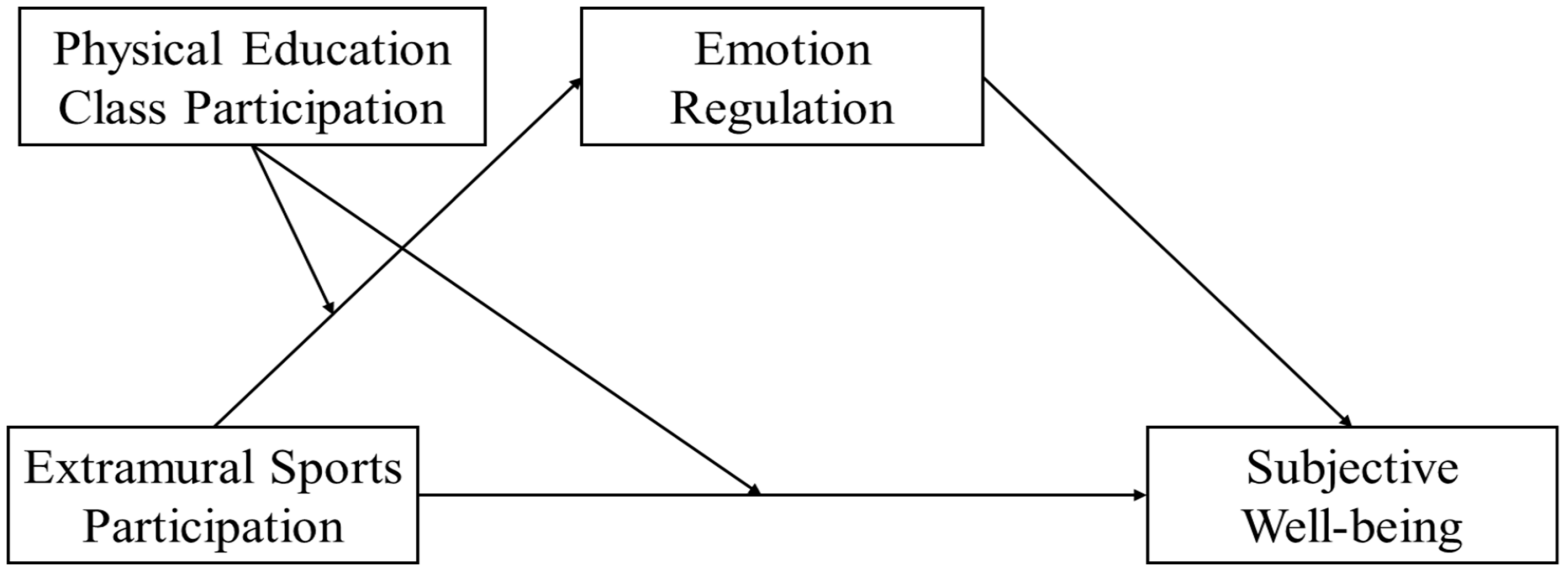
Hypothesized model diagram of the mediating role of emotion regulation and the moderating role of physical education class participation.

In summary, this study constructs a moderated mediation model from the perspective of individual-environment interaction theory (56), integrating the self-system model of sports participation and the self-regulatory executive functioning theory of emotion regulation (50, 57) (see Figure 1). It examines the relationships among extracurricular sports participation, emotion regulation, and the number of days students participate in PE classes per week, and their impact on subjective well-being. Specifically, this study investigates the mediating (emotion regulation) and moderating (number of days of PE class participation per week) mechanisms by which extracurricular sports participation predicts student well-being. The goal is to provide empirical support and theoretical guidance to clarify how extracurricular sports participation impacts student well-being, guide students to actively participate in sports, enhance emotion regulation abilities, and improve psychological health.

## 2 Materials and methods

### 2.1 Participants

This study is based on the latest PISA assessment results. The PISA 2022 assessment involved about 690,000 15-year-old students from 81 countries and regions, including 37 OECD member countries (2). After excluding invalid and missing data, we analyzed the mechanisms among extracurricular sports participation, emotion regulation, subjective well-being, and PE class participation for 113,203 junior high school students. Unlike previous PISA assessments, the PISA 2022 assessment was conducted during the global COVID-19 pandemic, focusing on both students’ academic performance and various non-cognitive learning outcomes.

### 2.2 Research tools

#### 2.2.1 Extracurricular sports participation

PISA 2022 measured students’ participation in school sports activities with two questions (2):

1. “How many days per week before school do you exercise or practice a sport (e.g., running, cycling, aerobics, soccer, skating)?”

2. “How many days per week after school do you exercise or practice a sport (e.g., running, cycling, aerobics, soccer, skating)?”

Response options ranged from “0 days” to “5 days or more,” with higher scores indicating greater participation in extracurricular sports activities. The questionnaire demonstrated good reliability with a Cronbach’s α coefficient of 0.78 in this study.

#### 2.2.2 Emotion regulation

PISA 2022 included a socio-emotional skills module in the student questionnaire that featured emotion regulation (2). This section comprised 20 items: 10 on stress resistance and 10 on emotion control. Responses were scored on a 5-point scale, with higher scores indicating stronger emotion regulation. The questionnaire demonstrated good reliability with a Cronbach’s α coefficient of 0.82 in this study.

#### 2.2.3 Subjective well-being

The PISA 2022 Subjective Well-Being Questionnaire (SWBQ) assessed students’ well-being with 23 items (2). Excluding demographic variables, items were scored on a 4-point scale, with higher scores indicating greater subjective well-being. The questionnaire demonstrated good reliability with a Cronbach’s *α* coefficient of 0.63 in this study.

#### 2.2.4 PE class participation

PISA 2022 included the question, “How many days per week do you have PE classes on average this school year?” in the SWBQ to measure the weekly participation in PE classes. Responses ranged from “0 days” to “7 days,” with higher scores indicating more frequent participation (2). Results showed that students participated in PE classes an average of 2.95 days per week, with 36% participating only once per week.

### 2.3 Data analysis

Statistical analyses were conducted using SPSS 27.0 and moderated mediation effects were tested with the SPSS Process v4.1 macro. Descriptive statistics assessed the demographic characteristics of middle school students. Correlation analysis explored the relationships among extracurricular physical activity, emotion regulation, subjective well-being, and participation in physical education classes. Hierarchical regression analysis tested the hypotheses of this study. All results were deemed statistically significant at *p* < 0.05.

## 3 Results

### 3.1 Common method bias test

The Harman single-factor test was used to examine potential common method bias. Results showed that 14 factors had eigenvalues >1. The first common factor explained 29.14% of the variance, below the critical standard of 40%, indicating no significant common method bias.

### 3.2 Correlation analysis

Descriptive and correlation analyses revealed that extracurricular sports participation was significantly negatively correlated with emotion regulation and significantly positively correlated with subjective well-being and PE class participation. Emotion regulation was significantly positively correlated with subjective well-being and significantly negatively correlated with PE class participation. Subjective well-being was significantly positively correlated with PE class participation (see Table 1).

**Table 1:** Descriptive statistics and correlation matrix of variables.

Variable	*M*	*SD*	Extracurricular sports participation	Emotion regulation	Subjective well-being	PE Class participation
Extracurricular sports participation	6.55	3.55	1			
Emotion regulation	30.90	6.92	−0.02^***^	1		
Subjective well-being	154.55	49.10	0.08^***^	0.14^***^	1	
PE class participation	2.95	1.46	0.14^***^	−0.05^***^	0.07^***^	1

^***^p < 0.001.

### 3.3 Testing the moderated mediation model

Following the procedures for testing a moderated mediation model, the bias-corrected percentile bootstrap method (Bootstrap = 5,000) with a 95% confidence interval was used, controlling for country, gender, and health status. Model 4 in the Process macro was used to test the simple mediation effect. Results indicated (see Tables 2, 3) that extracurricular sports participation significantly predicted subjective well-being (*β* = 0.61, *t* = 21.83, *p* < 0.001). When the mediating variable was included, extracurricular sports participation still significantly predicted subjective well-being (*β* = 0.58, *t* = 21.03, *p* < 0.001). Extracurricular sports participation significantly positively predicted emotion regulation (*β* = 0.04, *t* = 7.27, *p* < 0.001), and emotion regulation significantly positively predicted subjective well-being (*β* = 143.48, *t* = 45.09, *p* < 0.001). The direct effect of extracurricular sports participation on subjective well-being and the mediating effect of emotion regulation both had bootstrap 95% confidence intervals that did not include 0, indicating that extracurricular sports participation directly predicts subjective well-being and also does so through the mediating role of emotion regulation. The direct effect (0.58) and the mediating effect (0.03) accounted for 95.4 and 4.6% of the total effect (0.61), respectively.

**Table 2:** Testing the mediating role of emotion regulation.

Regression equation		Fit indices			Significance of coefficients	
Outcome variable	Predictor variable	*R*	*R^2^*	*F (d f)*	*β*	*t*
Subjective well-being		0.16	0.03	686.39^***^		
	Country				0.015	33.35^***^
	Gender				−2.59	−13.61^***^
	Health status				−3.11	−25.58^***^
	Extracurricular sports participation				0.61	21.83^***^
Emotion regulation		0.17	0.03	768.84^***^		
	Country				0.00	10.69^***^
	Gender				−1.57	−42.93^***^
	Health status				0.62	26.89^***^
	Extracurricular sports participation				0.04	7.27^***^
Subjective well-being		0.21	0.04	966.31^***^		
	Country				0.01	32.16^***^
	Gender				−1.46	−7.70^***^
	Health status				−3.55	−29.46^***^
	Emotion regulation				143.48	45.09^***^
	Extracurricular sports participation				0.58	21.03^***^

All variables in the model were standardized before being entered into the regression equation. ^***^p < 0.001.

**Table 3:** Total effect, direct effect, and mediating effect decomposition.

	Effect value	Boot SE	Boot CI lower limit	Boot CI upper limit	Relative effect value
Total effect	0.61	0.03	0.55	0.66	100%
Direct effect	0.58	0.03	0.53	0.64	95.40%
Mediating effect of emotion regulation	0.03	0.00	0.02	0.04	4.60%

Boot SE, Boot CI lower limit, and Boot CI upper limit refer to the standard error, lower limit, and upper limit of the 95% confidence interval estimated by the bias-corrected percentile bootstrap method. All values are rounded to two decimal places.

Next, Model 8 of the SPSS macro, assuming moderation in the first half of the mediation model and the direct path, was used to test the moderated mediation model, controlling for country, gender, and health status. Results indicated (see Tables 4, 5) that when PE class participation was included, the interaction term of extracurricular sports participation and PE class participation significantly predicted both subjective well-being and emotion regulation (subjective well-being: *β* = 0.07, *t* = 5.48, *p* < 0.001; emotion regulation: *β* = 0.02, *t* = 5.02, *p* < 0.005). This suggests that PE class participation moderates both the direct prediction of subjective well-being and the prediction of emotion regulation by extracurricular sports participation.

**Table 4:** Testing the moderated mediation model.

Regression equation		Fit indices			Significance of coefficients	
Outcome variable	Predictor variable	*R*	*R^2^*	*F (d f)*	*β*	*t*
Emotion regulation		0.17	0.03	519.13^***^		
	Country				0.00	9.57^***^
	Gender				−1.54	−41.70^***^
	Health status				0.62	26.22^***^
	Extracurricular sports participation				0.04	7.04^***^
	PE class participation				−0.14	−10.99^***^
	Extracurricular sports participation × PE class participation				0.02	5.02^***^
Subjective well-being		0.24	0.06	900.50^***^		
	Country				0.01	31.49^***^
	Gender				−0.89	−5.96^***^
	Health status				−0.35	−36.91^***^
	Emotion regulation				0.61	47.90^***^
	Extracurricular sports participation				0.62	28.35^***^
	PE class participation				0.50	10.01^***^
	Extracurricular sports participation × PE class participation				0.07	5.48^***^

^***^p < 0.001.

**Table 5:** Direct and mediating effects at different levels of PE class participation.

	PE class participation	Effect value	Boot SE	Boot CI lower limit	Boot CI upper limit
Direct effect	−1.44 (*M*−1*SD*)	0.52	0.03	0.46	0.58
	0.00 (*M*)	0.62	0.02	0.58	0.67
	1.44 (*M* + 1*SD*)	0.73	0.03	0.67	0.79
Mediating effect of emotion regulation	−1.44 (*M*−1*SD*)	0.01	0.01	0.00	0.02
	0.00 (*M*)	0.02	0.00	0.02	0.03
	1.44 (*M* + 1*SD*)	0.04	0.01	0.03	0.05

**Figure 2:**
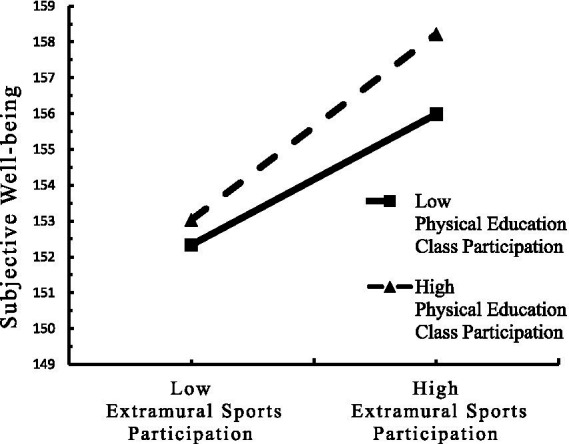
The moderating effect of PE class participation on the relationship between extracurricular sports participation and subjective well-being.

**Figure 3:**
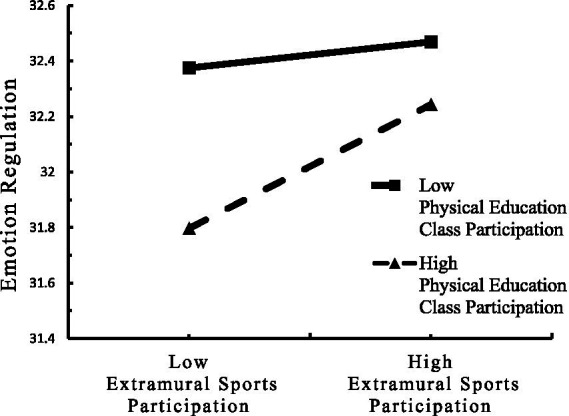
The moderating effect of PE class participation on the relationship between extracurricular sports participation and emotion regulation.

To clarify the trend of the moderating effect of PE class participation, scores were divided into high and low groups based on plus and minus one standard deviation. A simple slope test examined the predictive effect of extracurricular sports participation on subjective well-being at different levels of PE class participation. Results showed that for students with high PE class participation (M + 1SD), extracurricular sports participation significantly positively predicted subjective well-being (*β*simple = 0.73, *t* = 25.42, *p* < 0.001). For students with low PE class participation (M−1SD), extracurricular sports participation also significantly positively predicted subjective well-being, but the effect was smaller (*β*_simple_ = 0.52, *t* = 17.245, *p* < 0.001). This indicates that the predictive effect of extracurricular sports participation on subjective well-being increases with the number of days of PE class participation per week (see Table 5 and Figure 2). Similarly, for students with high PE class participation (M + 1SD), extracurricular sports participation significantly positively predicted emotion regulation (*β*simple = 0.06, *t* = 8.79, *p* < 0.001). For students with low PE class participation (M−1SD), extracurricular sports participation did not significantly predict emotion regulation (*β*simple = 0.01, *t* = 1.91, *p* > 0.05). This indicates that the predictive effect of extracurricular sports participation on emotion regulation increases with the number of days of PE class participation per week (see Table 5 and Figure 3). Furthermore, the mediating effect of emotion regulation in the relationship between extracurricular sports participation and subjective well-being increases with the number of days of PE class participation per week (58).

## 4 Discussion

Based on the self-system model of extracurricular sports participation and the self-regulatory executive functioning theory of emotion regulation (50, 57), and combined with existing research, this study constructs a moderated mediation model from the perspective of individual-environment interaction theory (56). The model clarifies how extracurricular sports participation influences students’ subjective well-being through the mediating role of emotion regulation and under what conditions this influence is more significant through the moderating role of PE class participation. The study’s results have important theoretical and practical implications for understanding the relationship between extracurricular sports participation and individual well-being. They guide students to actively participate in sports activities and enhance their emotion regulation abilities and life satisfaction.

### 4.1 The mediating role of emotion regulation

Emotion regulation is a crucial component of executive functioning (59). Exploring the mediating role of emotion regulation in the relationship between extracurricular sports participation and subjective well-being reveals how sports participation positively influences emotion regulation from a cognitive processing perspective and elucidates the mechanisms enhancing subjective well-being (58). This study found that extracurricular sports participation predicts students’ subjective well-being through the mediating role of emotion regulation (58). This finding supports previous research suggesting that sports participation directly enhances students’ subjective well-being and indirectly influences it through psychological capital, interpersonal relationships, self-esteem, cognitive reappraisal, and psychological resilience (10, 60, 61). Adolescence is considered the ideal time to encourage the development of positive emotions (41). Positive emotions, as components of mental well-being, are characterized by joy, enjoyment, and contentment, reflecting an individual’s overall subjective well-being (45, 46). Positive emotions help children and adolescents develop favorable personalities (62), organize interpersonal relationships (63), and solve group-related problems (41). This helps them maintain vitality and achieve a greater sense of well-being (64).Extracurricular sports participation provides a social interaction setting, enhancing emotion regulation abilities and thereby improving students’ subjective well-being. Positive emotions, as a component of psychological well-being, generally manifest as pleasure, enjoyment, and satisfaction, reflecting overall subjective well-being (45). Childhood and adolescence are ideal periods for developing emotion regulation abilities, contributing to the development of good personality traits (62), interpersonal relationships (63), and the resolution of group-related issues. This helps maintain vitality and achieve greater happiness (41, 64). Therefore, extracurricular sports participation enhances students’ subjective well-being by improving emotion regulation abilities.

### 4.2 The moderating role of PE class participation

This study constructs a moderated mediation model based on individual-environment interaction theory to examine the moderating role of PE class participation in the relationship between extracurricular sports participation, emotion regulation, and subjective well-being. Results indicate that PE class participation moderates both the relationship between extracurricular sports participation and subjective well-being and the first half of the mediation chain of “extracurricular sports participation—emotion regulation—subjective well-being.” Specifically, compared to individuals with high PE class participation, the direct effect of extracurricular sports participation on subjective well-being is more significant for those with low PE class participation. This finding indicates that the mechanism for enhancing subjective well-being (the mediating role of emotion regulation) exhibits individual differences and that PE class participation ensures other factors enhance subjective well-being (58). This is consistent with previous research findings (41, 44, 65). First, high PE class participation can improve students’ emotion regulation abilities (65). Additionally, sports can enhance psychological emotions, self-confidence, and overall well-being, making increased participation beneficial (6). Therefore, PE class participation positively promotes the effects of extracurricular sports participation and enhances subjective well-being. Secondly, the study found that for individuals with low PE class participation, the effect of extracurricular sports participation on emotion regulation is weak, reducing its impact on enhancing subjective well-being. This finding suggests that PE class participation moderates the influence of other variables on executive functioning (emotion regulation abilities). Previous research has found positive correlations between sports activities and various aspects of subjective well-being (66, 67), such as vitality, health-related quality of life, leisure satisfaction, and life satisfaction (58, 68, 69). Some studies suggest that these effects may be due to enhanced self-efficacy, self-esteem, or positive emotions, which are considered by-products of sports activities (70, 71). Therefore, high PE class participation more effectively promotes the positive impact of extracurricular sports participation on emotion regulation abilities and enhances subjective well-being. Unfortunately, studies show that a large proportion of adolescents do not engage in regular physical exercise, except for walking for at least 15 min daily (72). The percentage of adolescents who engage in daily physical exercise ranges from 10.3 to 26.7%, indicating that the proportion meeting daily physical activity recommendations is low (72–74).

## 5 Conclusion

The study results indicate that the model reveals how extracurricular sports participation enhances students’ subjective well-being through emotion regulation and shows individual differences in this mechanism moderated by PE class participation. This moderated mediation model explains how extracurricular sports participation influences students’ subjective well-being and under what conditions the effects are most significant. This is significant for promoting and deepening research on the relationship between sports participation and psychological well-being. The results indicate that emotion regulation is a key mechanism through which extracurricular sports participation enhances students’ well-being, moderated by the number of PE class days per week. This finding aligns with the educational value of physical education and health curricula and integrates the self-system model of sports participation with the self-regulatory executive functioning theory, promoting a comprehensive model for enhancing well-being.

Additionally, this model guides students to participate in sports for physical and mental health, reinforcing the positive impact on well-being. Data from the PISA 2022 survey indicate that 36% of students participate in PE classes only once per week on average, warranting attention. Schools should encourage students to participate in various sports activities, implement national policies, avoid prioritizing academic performance over mental health, and enhance students’ well-being. Schools should comply with national regulations on physical education, increase PE and health class hours appropriately, and create opportunities for students to participate in sports activities. Schools should avoid increasing class hours without ensuring quality, addressing the issue of students enjoying “sports activities” but not “PE classes.”

## 6 Limitations and future directions

This study has several limitations. First, data were collected during the COVID-19 pandemic, which may limit the sample. Second, the cross-sectional nature of the data limits causal inferences and trend analysis. Future research should adopt longitudinal designs or experimental studies, using multi-level models or manipulating independent and mediating variables to explore causal relationships between sports activities and subjective well-being. Finally, emotion regulation, a key component of self-regulatory executive functioning, has been a research focus, with relatively mature intervention paradigms (65, 75, 76). Future research can explore scientific experimental paradigms from the perspective of sports activities to further clarify the impact on emotion regulation and delve deeper into the role of emotion regulation in the relationship between sports activities and subjective well-being.

## Data Availability

Publicly available datasets were analyzed in this study. This data can be found at: https://www.oecd.org/pisa/data/.
